# Community Engagement Studios to advance multi-site research with older adults

**DOI:** 10.1017/cts.2024.630

**Published:** 2024-10-31

**Authors:** Meredith C. Masel, Kerri L. Cavanaugh, Sharon P. Croisant, Krista Bohn, James S. Goodwin, Martha L. Bruce, Paul J. Barr

**Affiliations:** 1 Department of Population Health & Health Disparities, The University of Texas Medical Branch School of Public & Population Health, Galveston, TX, USA; 2 Claude D. Pepper Older Americans Independence Center, The University of Texas Medical Branch, Galveston, TX, USA; 3 Sealy Center on Aging, The University of Texas Medical Branch, Galveston, TX, USA; 4 Vanderbilt Center for Effective Health Communication, Vanderbilt University Medical Center, Nashville, TN, USA; 5 Vanderbilt Center for Clinical Quality and Implementation Research, Vanderbilt University Medical Center, Nashville, TN, USA; 6 Department of Biomedical Informatics, Vanderbilt University Medical Center, Nashville, TN, USA; 7 Division of Nephrology & Hypertension, Department of Medicine, Vanderbilt University Medical Center, Nashville, TN, USA; 8 Department of Epidemiology, The University of Texas Medical Branch School of Public & Population Health, Galveston, TX, USA; 9 Health Education and Translational Research Engagement, Institute for Translational Sciences, University of Texas Medical Branch, Galveston, TX, USA; 10 Department of Internal Medicine, University of Texas Medical Branch, Galveston, TX, USA; 11 Department of Psychiatry, Geisel School of Medicine at Dartmouth, Hanover, NH, USA; 12 The Dartmouth Institute for Health Policy & Clinical Practice, Geisel School of Medicine at Dartmouth, Hanover, NH, USA; 13 The Center for Technology and Behavioral Health, Geisel School of Medicine at Dartmouth, Lebanon, NH, USA; 14 Department of Biomedical Data Science, Geisel School of Medicine at Dartmouth, Lebanon, NH, USA

**Keywords:** Community Engagement Studio, RCT, multi-site, communication, audio recording

## Abstract

**Introduction:**

Operationalizing multi-site Community Engagement (CE) Studios to inform a research program is valuable for researchers. We describe the process and outcomes of hosting three CE Studios with Community Experts aged 65 years or older with chronic conditions and care partners of older adults. Experts gave feedback about processes for testing the feasibility, efficacy, effectiveness, and implementation of audio recording clinic visits and sharing recordings with patients who have multimorbidity and their care partners.

**Methods:**

The CE Cores of the Clinical and Translational Science Awards Programs at three academic health science centers created a joint CE Studio guide. Studios were conducted iteratively by site. Following receipt of the final reports, responses were compared to find themes, similarities, and differences on four topics in addition to overall commentary: Recruitment and Retention, Study Protocol, Study Reminders and Frequency, and Recording Technology.

**Results:**

Eighteen older adults and care partners in three states provided valuable feedback to inform multi-site trials. Feedback influenced multiple aspects of trials in process or subsequently funded. Experts provided critique on the wording of study invitations, information sheets, and reminders to engage in study procedures. Experts were concerned for participants being disappointed by randomization to a control arm and advised how investigators should prepare to address that.

**Conclusions:**

Multi-site CE Studios should be consecutive, so each team can learn from the previous teams. Using the CES Toolkit ensures that final reports were easily comparable and utilized to develop a research program that now includes three federally funded clinical trials.

## Introduction

Despite increasing dialog about the audio recording of healthcare visits, including in primary care, many questions about its impact and implementation impede its widespread adoption. Some healthcare providers and patients routinely audio-record in the clinic[1]. Audio recording has been associated with greater patient satisfaction and understanding of the information from the visit in the short term[2], but the research is limited by short-term observation periods, one-off recordings of a single visit, settings representing only one healthcare site or system, the type of recording equipment, and a focus on specialty disease conditions such as surgery or oncology. To significantly advance our research program about understanding of the role of routine audio recording among older adults living with multiple chronic conditions, we sought to be authentically informed by the people who will benefit from the research.

Authentic inclusion of patient stakeholders in the process of research means soliciting and using community members’ thoughts, opinions, and recommendations to develop and carry out research. The Community Engagement (CE) Studio, designed by the Meharry-Vanderbilt Community-Engaged Research Core beginning in 2009, has emerged as an effective vehicle to link researchers with community members, also known as Community Experts, who provide valuable input to research teams[3]. Utilizing CE Studios, versus not engaging the community, has been associated with funded research proposals, greater numbers of manuscript publications, and an increase in the feasibility of study designs[4]. They provide benefits to Community Experts who participate, with over 99% of Experts saying their experience was positive and that they would do it again[4]. There are no expressed disadvantages to engaging community members in research planning[5].

**Figure 1.**
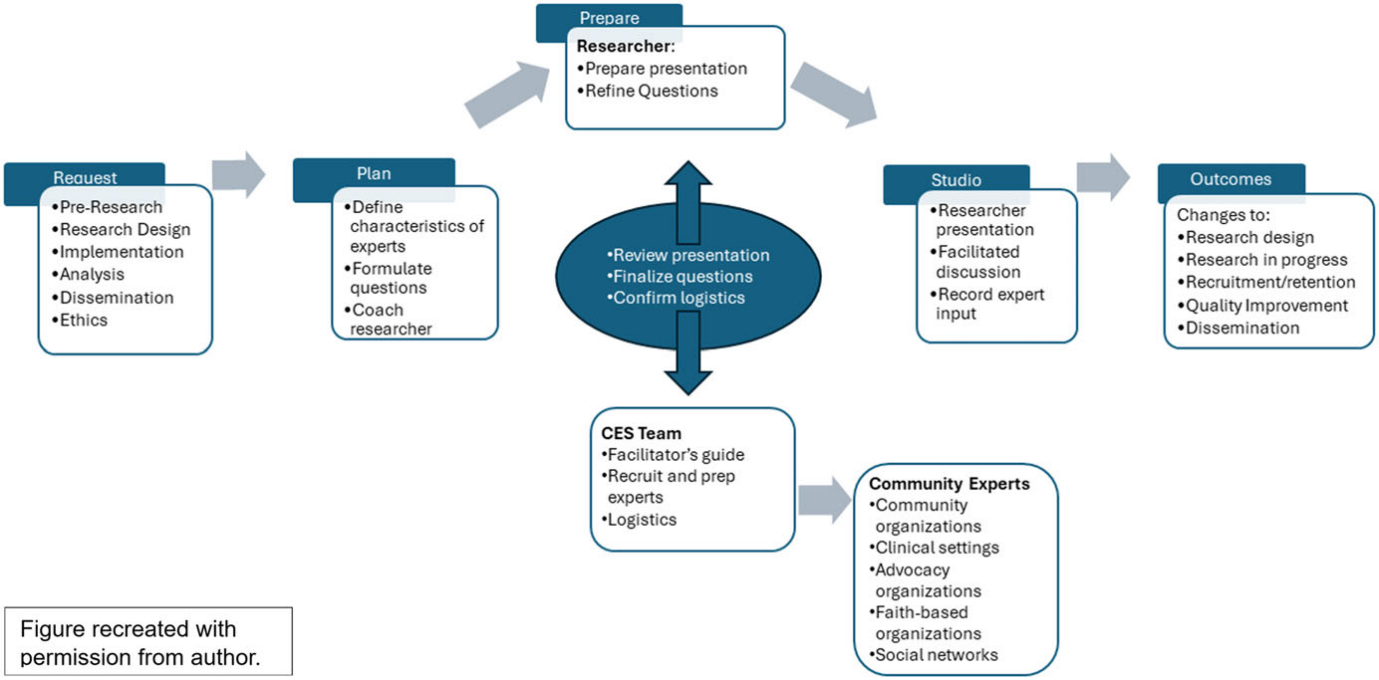
Community Engagement Studio Process (CES 2.0 Toolkit Page 16) [6].

CE Studios require collaboration among Community Experts, a research team, and a CE Studio team separate from the research team (Fig. 1). A CE Studio team invites representatives of a proposed study population to a community-based location where they get acquainted, listen to an overview of the research proposed, and provide feedback on specific questions from researchers for designing research programs. This effort to incorporate the voice of the “end user” into a research plan has been successfully applied for over a decade and allows researchers to gather significant feedback efficiently[4]. Our objectives are to describe the design of CE Studios conducted in three states and the outcomes on planning for multi-site research about the use of audio recordings with older adults and their primary care providers.

## CE Studios in support of theaudio pilot trial and proposals thereafter

When the studios were conducted, the research team, consisting of three principal investigators at Dartmouth College (Hanover, NH), The University of Texas Medical Branch at Galveston (Galveston, TX), and Vanderbilt University Medical Center (Nashville, TN), had begun a trial of feasibility and acceptability titled, “The effect of clinic visit audio recordings for self-management in older adults,” (AUDIO Trial) funded by the National Institutes on Aging (R56AG061522). By that time, the team was preparing proposals for larger, phase III trials. The CE Studios were conducted to both 1) refine the pilot trial materials and methods if possible and 2) genuinely invite Expert input as we prepared materials and methods for phase III proposals with the intervention. Materials and methods included recruitment and retention plans, randomization, participant compensation, and patient facing materials (i.e., participant intervention reminder emails).

The protocol and methods presented to the Experts in the studios were from the feasibility trial. In sum, investigators proposed recruiting 90 adults aged 65 years and older who attended primary care for management of multimorbidity including both hypertension and diabetes. Participants would be randomized to usual care (receiving an after-visit summary based on the standard at all sites) or to receive audio recordings of all their primary care clinic visits during a 3-month period, accessed via a secure online platform. Participants in the audio arm would receive listening reminders via email. Participants in both arms would complete surveys on topics such as demographics, self-management, provider communication, and adherence at multiple time points, including after interim visits. The phase III proposals had similar aspects to the pilot in process such as the audio intervention, the longitudinal data collection, participant age, and participant multimorbidity.

All the information gathered from the CE Studios was reviewed by the investigators and incorporated into the pilot and in the proposals for two larger phase III trials involving audio use among older adult patients with multimorbidity. Despite the ideal scenario of conducting CE Studios prior to writing any protocols, the investigators kept an open mind about including feedback wherever possible in the R56 AUDIO Trial pilot that was in process.

## Materials and methods

We followed the procedures as recommended by the authors of the “CES Toolkit 2.0” as shown in Fig. 1 [6]. A CE Studio is not a research activity and is not governed by an Institutional Review Board. Although comments and quotes are collected, the studio format is different from qualitative data collection in that it is intended to be a bidirectional conversation. Comments are not analyzed using qualitative methods. Conversational content is solicited and summarized for researchers by a separate CE Studio team. Then, recommendations are applied by researchers to research protocol and design development [6].

### Setting

Teams at each of the three sites collaborated to develop a single CE Studio format and devise a series of questions for local Community Experts who mirrored our target research population. The question list was designed by the teams to inform the multi-site research program about the routine sharing of primary care visit recordings online with older adult patients managing multimorbidity and their care partners. Each institution had a National Institutes of Health Clinical and Translational Science Award (CTSA) that provided the infrastructure to support the studios through their respective Community Engagement Cores [UL1TR001086, UL1TR001439, UL1TR002243].

### Identifying Community Experts

We first identified a local community navigator, a person trusted within the community to help seek out Community Experts at each of our sites. We aimed to involve 8–10 Experts per site as recommended by the Toolkit [6]. Various modes advertised studios to Community Experts. Advertising included the basic structure of the studio (meal, presentation, question, and answer session), the time required, the $50 participant compensation, and the location. The VUMC CE core, with a longer-standing studio program, used a database of Community Experts with an interest in certain topic areas and contacted relevant Experts directly through their preferred mode of communication. In the two other CE Studio programs, which were newer and had fewer Expert members, a broader approach was used by advertising among listservs of community groups of older adults, such as the Dartmouth Centers for Health and Aging, a community group that provides resources and support to older adults, as well as The Osher Lifelong Learning Institute and via word of mouth at UTMB Health.

Community Experts chosen to advise this research design could be of any racial/ethnic group or gender. We required that Experts were similar to our target participant inclusion for our pilot trial and potential subsequent trials that included care partners. Experts were either aged 65 years or older with at least one chronic health condition or that they were care partners (e.g., spouses or adult children) of community-dwelling older adults with at least one chronic condition. Once selected, Community Experts received a reminder about the event (see **Supplemental file** 1).

### Studio Format

The decision on when (i.e., time of day) and where to have the studio was determined with a community navigator. Community Experts in each studio by location met separately but knew about the two other groups of Experts from the other sites that would also be contributing. We completed studios sequentially, creating an efficient iterative feedback process, informing the conduct of the following studios. The studio at Dartmouth happened first, resulting in modifications to the subsequent PI introductory presentations. Specifically, during the topic introduction at the first studio when Experts learned about audio recording an entire clinic visit, they spent excess time discussing the idea of adding a transcript to the intervention which would have expanded the study scope beyond the ability of the study team. Due to the potential for error in transcripts, this was not under consideration by the study team and therefore was clarified by investigators at the beginning of the subsequent studios.

The first two studios had a similar structure and occurred in person. A Principal Investigator joined the community navigator and a CE staff member at the University. Community Experts gathered in a centrally located community center and restaurant, respectively, and shared a meal prior to commencing the feedback activities. Unexpectedly, due to health and safety precautions for COVID-19 enacted in early March, the third studio at Vanderbilt University Medical Center was held via Zoom.

### Studio content

After getting acquainted, the site Principal Investigator gave a 10-minute PowerPoint presentation about the specific problem the proposed research studies aimed to address: to determine the effectiveness of audio recording of clinic visits in terms of the ability of patients to self-manage their healthcare. They reviewed initial research plans, including treatment and comparison arms, follow-up survey schedules, listening regimes for the audio recordings, and survey delivery modes (e.g., in person vs. online vs. over the phone). After the presentation, the Principal Investigator handed over the studio to a facilitator and provided input if asked by the Community Experts. The facilitator was necessary to create a neutral, supportive environment that promoted open, transparent discussion, without interjecting personal views. The facilitator guided the group through discussions of four main topic areas: Recommendations for Recruitment and Retention, Study Protocol, Study Reminders from study staff, Recording Technology, as well as overall thoughts on usefulness of audio recording visits (**Supplemental File** 2). As recommended by CE Studio guidelines, the studios were not audio-/video-recorded and notes were captured in real time by a note taker on a board visible to the Community Experts.

### Obtaining feedback

Discussion moderation was conducted by a trained facilitator who met with the Site Principal Investigator in advance to review the aims of the studio. The facilitator encouraged Community Experts to give their initial reactions and ask any questions that arose after listening to the investigator’s presentation. Community Experts were asked to share their honest opinions, good or bad, as their feedback was critical to ensure the highest likelihood of completing a successful project. Following the general discussion, the facilitator asked a series of questions about the potential benefits of the research, concerns, and the usefulness of audio recording technology to them or to people in their community.

The facilitator ensured the activity occurred within the promised time limit and covered all areas of input that the research team had requested from the Community Experts. The facilitator served as a moderator to give space for participants to share differing views while maintaining a collaborative atmosphere. The Site Principal Investigator was present during the discussion to answer questions from the Community Experts, but otherwise was removed from participation or comment; this strategy is to reduce possible bias or undue influence of the investigator on the opinions and views of the Experts. After the studio, the CE university staff and navigators prepared final summary reports for each institution’s research team. The report was a standardized document with a summary of key findings and direct quotes/comments from Community Experts captured by a note taker during the studio.

## Results

Between March 2 and March 20, 2020, investigators held three 90-minute CE Studios. In total, 18 Community Experts participated across the three research locations (6 per site) (see Table 1). The Experts, 6 males and 12 females, were of white, black, or Hispanic race/ethnicity. The majority of Community Experts (89%) were aged 65 years or over. At least two Experts were there as care partners of people with chronic conditions and the remaining filled both roles (patient/care partner). Table 2 provides examples of the questions researchers asked and the responses from Community Experts. Below is a synthesis of the feedback obtained from each of the three studios on the main topic areas, and the impact of that feedback on trial planning and the multiple Principal Investigators’ research program. Generally, Experts had similar opinions. When they did not, investigators followed our pilot grant leadership plan which was to discuss as a team the pros and cons of making changes and, in the event of disagreement, the contact PI would have the final decision.

**Table 1.** Demographic characteristics of 18 participants in the Community Engagement Studios across three sites

Community Expert’s Characteristics	DH	UTMB Health	VUMC
Gender			
Male	2	2	2
Female	4	4	4
Age (years)			
<65		1	1
65+	6	5	5
Race/Ethnicity			
White	6	4	4
Hispanic		1	
Black		1	2

*Note*: DH = Dartmouth College; UTMB Health = University of Texas Medical Branch; VUMC = Vanderbilt University Medical Center.

**Table 2.** Sample of interview questions, Community Expert responses, and actions taken by the study team in response to feedback from multi-site Community Engagement Studios

Topic/sub-topic	Interview guide question (examples)	Community Expert responses	Research team actions
**Recruitment**	How would you prefer to hear about a study like this? How do you think people in your community will react to being in the [usual care] group?	“If the physician asked, I’d be more likely to say yes.” “I think it could hurt some people’s feelings to be in the control group.”	Ensured that potential participants received a note from their provider about the study and that the provider was comfortable with their participation. Prepared study staff to be able to clarify that participation was voluntary in the case a person was randomized to an arm other than the one they wanted.
**Materials**	Regarding reminders for study activities, what text would be most impactful or motivating for you and people in your community How can we make the reminder most motivating for you to review you recording?	“Need to make sure it’s not spam; it sounds like spam.” “Sender matters – need to recognize or won’t open” “Email 3: rephrase as final “opportunity” to visit recording. More personable and more inviting”	Changed the wording of automatic reminders to be more personable and reflect specific suggestions from the Experts. Ensure that study reminders come from an email address the patient is familiar with or has been prepared to look for.
**Implementation**	Recap technology. How do you think this might work for YOU and people in your community? How would a recording of your doctor’s visit be useful for you?	“Can’t picture the advantage of a recording because I am a very visual person, like another participant.” “Technology use in elderly populations needs to be easy.” “I think this is an awesome project, because my parents are 60 and 65 and my mom’s response is always “they didn’t say anything” when I ask about the Drs visits.”	Performed field testing and a pilot trial to continuously improve the user interface for the intervention. Expand the research program emphasis on care partners.
**Other**	“What would you like to see the researcher do differently?”	“Add paper transcription/translation.” “[Provide access] for other languages besides English to help clarify clinic visit information.”	The intervention user interface was translated along with all final study materials. We continuously work to expand access and recruitment of Spanish-speaking study participants.

Figure recreated with permission from author.

### Recruitment and retention

Community Experts commented on the mode of initial contact. Experts shared ideas including placing flyers in waiting rooms, advertisements in local newspapers, and community talks. At all sites, Experts suggested that advertisements or community talks would be fewer effective methods and recommended that we utilize trusted relationships with primary care doctors/nurses as the point of initial contact. One participant stated, *“If the physician asked, I’d be more likely to say yes.”*

### Research team actions

We recruited healthcare providers first and then sent initial contact letters signed by the providers, inviting potentially eligible patients to consider the research study.

Community Experts reviewed the research team’s recommendations for study participant compensation. We proposed that all participants receive the same amount of compensation whether they were in the group receiving audio recordings or in the usual care control group. Experts’ opinions ranged from cautioning that the study would be a lot of work, to feeling like any token of compensation would be appreciated. Community Experts at Dartmouth cited other activities in the community that offered more compensation for less time and/or effort.

### Research team actions

No changes made to the participant compensation plan to avoid any chance of coercion. We also decreased survey burden in subsequent trial designs.

Community Experts were asked about barriers they could envision during the recruitment process. In general, internet savviness and access were the main concerns across all three sites. Experts were also asked, “*This is a randomized experiment. That means people won’t get to choose which group they are in. How do you think people in your community will react to being in the group that gets the recordings? How do you think people will react to being in the other group?*” Concerns were shared at VUMC and UTMB Health about being randomized to the control arm: *“I think it could hurt some people’s feelings to be in the control group.”* and *“Will there be a 3rd group for those who WANT to be in the recording group but aren’t assigned?”* Those at Dartmouth believed randomization to either arm was not a concern because treatment would not be affected. Community Experts made specific recommendations to the research teams about preparing potential participants for randomization, such as making sure that people randomized to control knew their enrollment was a valuable contribution: *“It’s all about how you tell them that they won’t be in it. It should be presented in the right way-be sure that they know that this is helping research and improving practice.”*

### Research team actions

Recruitment scripts and consent form language were modified to include Community Expert recommendations about how to set expectations regarding randomization assignment, scientific equipoise, and the equal value of all study participants’ contributions. Coordinators practiced enrollment procedures, including how to manage the possibility of a person being disappointed with their assigned arm. Regarding concerns about being randomized to usual care, our study teams were prepared to allow anybody to decline to participate and speak to their doctor on their own about recording.

#### Study protocol

All sites endorsed the overall trial design comparing audio recording to usual care as a good idea. For example, one Community Expert said that a recording would be an improvement over the current after visit summary. Another said, *“I can’t see a disadvantage to this [project].”* A care partner mentioned, *“I think this is an awesome project because my parents are 60 and 65 and my mom’s response is always “they didn’t say anything’ when I ask about the doctor’s visits.”* At UTMB Health, Community Experts strongly believed that the trial itself and results could be particularly helpful for persons with dementia and care partners. Those Experts also asked if we could accommodate non-English speakers.

Community Experts questioned whether doctors or nurses would agree to do the trial and wondered about legal implications: *“What would prevent a patient from posting recording on social media or using to sue? How to protect doctor?”* A few Experts discussed the possibility that a patient or provider might act differently due to the presence of an audio recorder.

We also received suggestions for involving care partners. Community Experts at VUMC noted that it would be helpful for care partners to have the option to easily listen to recordings and that we should let participants know all the types of care partners with whom they could share.

### Research team actions

The comments informed our decision to remove a diagnosis of dementia as a hard exclusion criterion in our trials. Instead, we employed a six-item screening test^7^ to determine if the patient had the cognitive capacity to consent to participation, regardless of the existence of a diagnosis related to dementia. Discussions about non-English-speaking participants further influenced our study design. Processes have been developed to ensure ability to include medical interpreters, Spanish-speaking staff, and translated materials. Finally, all sites agreed to explicitly request that participants feel free to share recordings with anybody they consider a care partner, but not post recordings on social networking websites.

### Study reminders and frequency

Several types of study text-based reminders were presented to the Community Experts. For audio arm participants, the Experts reviewed drafts of automated emailed reminders that prompt them to listen to their recording and discussed the frequency and content of REDCap survey emailed reminders. Concerns were expressed about email volume in general and the possibility of SPAM filters causing emails to be hidden.

#### Reminders to Listen

For the audio arm, we proposed that participants would be asked to listen to their recordings at least twice, using system-generated email reminders. After reviewing email drafts and plans during the studio, Community Experts recommended changes in wording to phrase the availability of their recording as an *opportunity* for participants, and that listening was a *request* by the study team, rather than a demand or requirement. Feedback was given on action instructions within the email message as well: *“…clear that they should click the link if they want to access recordings.”*

At multiple sites, Community Experts suggested making sure the reminder emails had the name of the participant and the name of the healthcare provider associated with the recorded visit. They also suggested the reminder email come from a recognizable name, made recommendations for the subject line title, and suggested moving the web link to the recording closer to the top of the email: *“[it should say] Even though we understand that you did initially, we are REQUESTING that you do it a second time.”*

#### Research team actions

Following the Community Experts’ counsel, the wording of the email reminders and their content structure were improved for clarity and usability. Importantly, personalization of the messages was achieved through including their name in the salutation. While one study was limited to email reminders, future study iterations give study participants the option of receiving text to their personal cell phone, email reminders, or both modes of communication.

#### Reminders to Complete Surveys

As all study participants would be required to complete surveys, we asked Community Experts about the formatting of reminders (email and/or text). We again heard concerns about email overload. One Expert suggested adding a reminder about compensation as a motivating factor. They shared advice to ask participants how they preferred to receive reminders and Experts at UTMB Health also suggested offering phone call reminders. *“The best plan would be to ask the patient what their preferred communication style is and pick which one works best for the individual. Or use a combination technique (i.e., send reminder email AND phone call).”*

Research team actions

We took the advice of our Community Experts and trained study coordinators on multiple ways to communicate with participants after enrollment. Participants are provided a complete customized schedule of planned follow-ups that are included in the study design. For example, “Your next set of surveys is due on [date]. You will begin receiving reminders on [date] at [email] and/or [phone number].” The study coordinator maintained a document of the same written schedule of follow-ups to ensure they used the preferred methods when contacting participants to remind them about study surveys. Participants who preferred completing surveys over the phone were provided copies of the survey to keep at home and refer to while on the phone with the coordinator. This procedure was noted to be particularly helpful for those hard-of-hearing. It allowed them to follow along, and it ensured that they answered the exact same questions as those who filled out online surveys.

#### Recording technology

Community Experts endorsed the technology required for the trial but had concerns about their loved ones’ or their own ability to use the software. In addition, they shared concerns about the ability of a participant to hear their own recording and made suggestions about recorder placement in the exam room during recording. Experts strongly urged to systematically train participants in using the software: *“not optional even if they say they don’t have questions.” “Research coordinator could guide patients to computers and make sure they’re comfortable.” “Good training for programmers and participants in-person.”* Providing a tutorial/prep video, in addition to written instructions, was also suggested. *“It could give the patient time to think of questions.”*

Research team actions

We created a training video, as well as a study checklist to be completed by the coordinator, ensuring that all participants viewed the video, were trained, and demonstrated understanding of the steps to take to listen to their recordings. We also created a handbook to accompany the video that is given to the participant with all other study materials. We conducted field testing of recorder placement in the various clinic settings and, where applicable, utilized easy-to-find small rolling instrument trays to hold the recorders near the conversation. These trays could also be easily moved out of the way if necessary, such as during an exam.

## Discussion

Despite some examples of success, little has been fully described about operationalizing CE Studios to inform multi-site research where researchers seek patient groups from different geographic locations, where local attitudes may differ [8]. In this case, the Community Experts provided critical input about the study design, including: how to make the research welcoming to people like themselves; preferences about initial contact, compensation, and other participant engagement tactics; consent forms and survey structure (e.g., length, topics, and frequency); the process of revealing the study arm assignment to enrolled participants; drafts of text and email reminders; and suggestions for maximizing the benefit of a recording activity. The studios described influenced the processes or design of our originally funded pilot trial resulting in two federally funded phase III trials.

This is one of the few examples of multiple-site CE Studios and an early example of conducting a CE Studio virtually in 2020 due to coinciding with the beginning of the COVID-19 pandemic. Since then, one multi-site team from North Carolina, South Carolina, and Georgia reported in an oral presentation at an Academy Health Annual Research Meeting that they successfully developed a virtual CE Studio model to engage hard-to-reach rural participants with diabetes and their care partners in 2021 [8]. In 2022, Stock and colleagues from the New York State Center of Excellence for Alzheimer’s Disease (CEAD) elaborated that, even with the challenges faced by a need to conduct virtual studios during the pandemic, such as difficulty assessing participant’s body language, older adults in their community could participate with the help of center staff and provide great insight [9]. Our experience similarly demonstrated that the virtual mode of convening older adults is feasible and yields transformative information like our in-person studios.

Our findings are consistent with others that holding multiple CE Studios on the same topic can be fruitful and have a large impact on a research program. For example, in the STEP Together study, the virtual CE Studios yielded significant modifications to their effectiveness trial of incentives to engage in physical activity [10]. Their Community Experts in and around Philadelphia suggested changing the age range and adding a survey. They sought feedback on designing clinical trials to ensure accurate representation of participants in the local community. They learned ideas for better advertising phrasing (e.g., using the words “helpful conversations” or “chats” instead of the word “therapy”) as well as new places to post recruitment materials, such as in local pharmacies. They held six studios with adults aged 18 years and older and in their second round, they added a question to their facilitator guide. We also found it very useful to employ a consecutive approach, as opposed to conducting studios simultaneously. For example, immediately following the introductory presentation, some of our first studio participants were very interested in adding a study arm or extending the intervention to include a full transcript. That discussion at the site lasted for a long time, which depleted time intended for discussion about other topics. Therefore, it was helpful for the other sites to address this issue during the introductory presentation. At the second studio, despite addressing up front that we would not be able to provide transcripts which can be more prone to errors due to vocal cadence, accents, and recording quality, Experts were still interested in discussing it. We saved significant time, however, bringing it up first. This also informed the third studio staff to prepare for the potential discussion. Like other process improvement approaches, revising the plan for the studio itself based on initial feedback can enhance the depth and quality of the information gained from the Community Experts.

## Limitations

As we experienced, convening a studio posed a challenge when an organization was in the beginning stages of creating Community Expert databases. A Community Expert database contains information about members of the community who formally state (via an information form) that they have interests in and willingness to provide feedback to research teams on various topics. CE Core staff actively seek opportunities to reach diverse community members and solicit their willingness to serve in this capacity [6]. It takes significant time to build databases with enough members to support a wide variety of research topics. Due to the immaturity of two of the CE Studio databases at Dartmouth and UTMB, there were fewer Experts to contact resulting in a “first come, first served” recruitment approach. This was somewhat mitigated by our broad trial inclusion criteria allowing us to identify Experts quickly. Experts from a well-established database are more likely to have experienced the format and developed a comfort level with providing direct feedback in a group of strangers. Community Experts and investigators at UTMB and Dartmouth had less experience than those at Vanderbilt University Medical Center which may have contributed to the length of time spent on the very first issue raised about transcripts. At least one confirmed Expert per site did not attend the studio resulting in attendance lower than our target of 8–10 Experts per studio. We did not account for this possibility and should have searched harder for 10–12 Experts as recommended by the Toolkit [6].

Conducting multiple CE studios to inform a multi-site trial design for older adults was successful, with a large impact on the research program. We recommend the Community Engagement Studio Toolkit 2.0 [6], which provides a template and useful suggestions for building relationships with community organizations and individuals to invite them to participate in informing research. These findings contribute to the growing body of literature on gathering community members’ (from the target population) opinions on research involving older adults. This is a worthwhile effort for research teams or those planning intervention programs and an opportunity for older Community Experts to authentically and significantly lend their wisdom to research.

## Supporting information

Masel et al. supplementary material 1 Masel et al. supplementary material

Masel et al. supplementary material 2 Masel et al. supplementary material

## Supplementary material

The supplementary material for this article can be found at https://doi.org/10.1017/cts.2024.630.
