# Supplementary material for: Community Engagement Studios to advance multi-site research with older adults
Source: J Clin Transl Sci. 2024 Oct 31;8(1):e186. doi: 10.1017/cts.2024.630 (PMC11626571; doi:10.1017/cts.2024.630)

Supplemental File 1: Sample email confirmation used to prepare and remind community experts about the event


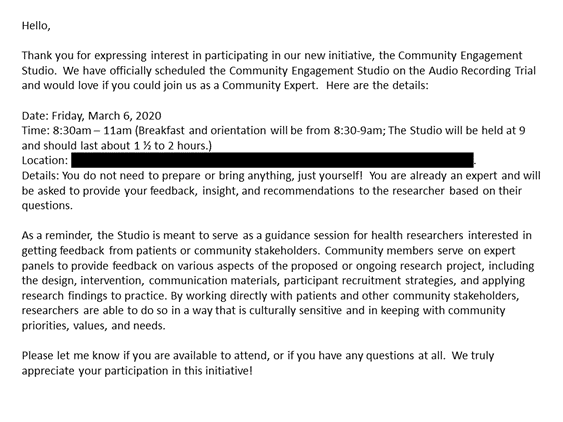

Supplement: Masel et al. supplementary material 1 — Masel et al. supplementary material [file S2059866124006307sup001.docx]
