# Supplementary material for: Community Engagement Studios to advance multi-site research with older adults
Source: J Clin Transl Sci. 2024 Oct 31;8(1):e186. doi: 10.1017/cts.2024.630 (PMC11626571; doi:10.1017/cts.2024.630)
Supplement: Masel et al. supplementary material 2 — Masel et al. supplementary material [file S2059866124006307sup002.docx]

**SUPPLEMENT 2**

**Community Engagement Studio**

**Facilitation Guide**

*[PI] Recording project studio*

*[Month] 2020*

*Welcome.*

*Introduce studio.*

*Are there any questions before we begin?*

First, introduce yourselves and tell us what superpower you wish you had.

[PI] presents (10 minutes)

**Research Plan**

- What’s your initial reaction to this project?
- What other questions do you have about the project?
- This is a randomized experiment. That means people won’t get to choose which group they are in.
  - How do you think people in your community will react to being in the group that gets the recordings?
  - How do you think people will react to being in the other group?

**Show video and distribute written instructions for recordings**

- These are the instructions for accessing recordings for your visit.
  - What do you think about these instructions?
- How could they be improved or more clear for people in your community?

**Text Reminders and frequency**

- Recap plan for text reminders.
  - What are your thoughts about this language?
  - What might you change?
- What is the right timing for text reminders?
- What text would be most impactful or motivating for you and people in your community?

**Recommendations for recording technology**

- Recap technology. How do you think this might work for YOU and people in your community?
- How would you, caregivers or family use the recordings?
- What might make the recordings work better for you?
- How might the medical conditions you have impact the recordings or how you plan to use the recordings?

**Recommendations for Recruitment**

- What will be the barriers for people like you to participate in this study?
- What would help people in your community participate?
- How would prefer to hear about a study like this?
  - Recap recruitment plan.
- What recommendations do you have for recruiting people into this study?
- What concerns would have to be resolved in order for you to participate?

**Surveys**

- This project will involve a number of surveys.
- Recap plan.
- What are your preferences for completing surveys online, over the phone or in person?
- How long is too long for a survey?

**What do you wish I asked?**

*Thank everyone for contributing. Feel free to use the notes page to tell us anything else you think we should hear.*

*Complete the feedback form.*

*We have gift cards to thank you for your participation.*
